# Analgesic and physiological effects in conscious sedation with different nitrous oxide concentrations

**DOI:** 10.4317/jced.52034

**Published:** 2015-02-01

**Authors:** Neus Bonafé-Monzó, Juan Rojo-Moreno, Montserrat Catalá-Pizarro

**Affiliations:** 1 Associate Professor of Pediatric Dentistry. University of Valencia, Spain; 2 Full Professor of Psychiatry. University of Valencia, Spain; 3 Full Professor of Pediatric Dentistry. University of Valencia, Spain

## Abstract

Objectives: to study the physiological changes, as well as the psychosedative and analgesic effects of nitrous oxide, in experimental conditions. Study Design: 101 dental students volunteers participated in a single nitrous oxide sedation session without dental treatment. Signs and symptoms were registered during and after the procedure. Pulse rate and hemoglobin oxygen saturation were monitored at: 100 per cent O2, 30 per cent N2O, 50 per cent N2O and 5 minutes after 100 per cent O2. A Likert scale was used to evaluate pain perception. The analgesic effects of nitrous oxide were evaluated at: 30 per cent N2O, 50 per cent N2O, and five minutes postoperatively. Results: Pulse rate and hemoglobin oxygen saturation decreased significantly through all the procedure and after recovery. However, oxygen saturation recovered after the final oxygenation. Only 8.2% of subjects reported the pain stimulus as being quite annoying when they inhaled 30 per cent N2O, while this percentage was of 15.8 % when inhaling 50 per cent N2O, and of 32.7 % during the recovery period. The most common effects of nitrous oxide sedation were bright eyes (99%), voice change (98%) and smiling (91%). Most of the subjects reported tingling (98%) and relax (91.1%) Conclusions: nitrous oxide causes a significant decrease in heart rate and oxygen saturation, but always within safety limits. Maintaining an appropriate level of consciousness was confirmed as a feature in 50 per cent dose in this study. The analgesic effect of nitrous oxide was confirmed but a dose dependency could not be established.

** Key words:**Nitrous oxide, conscious sedation, anxiolysis, safety, physiogical parameters, signs, symptoms, analgesia.

## Introduction

Dentistry is nowadays accessible to a great part of the population regardless of their idiosyncrasy or social stratum, but fear of pain and rejection to stress situations make sometimes difficult to provide quality dental care.

Prevention as well as control of pain and stress associated with therapeutic procedures, enable unhealthy people to improve well-being. Therefore, it is not surprising that patients are increasingly demanding, for them and their children, not to experience pain or stress in dental procedures. This fact would explain the remarkable increase of the use of conscious sedation in dental clinics, specially through nitrous oxide-oxygen inhalation. Conscious sedation is a drug-induced depression of consciousness during which patients responds to verbal commands, either alone or accompanied by light tactile stimulation. No interventions are required to maintain a patient airway, and spontaneous ventilation is adequate. Cardiovascular function is usually maintained (1).

Nitrous oxide is a colorless and odorless gas, hardly visible and with a sweet taste, which presents a low tissue solubility. Among its effects, the most important is the decrease of pain and anxiety, although it cannot substitute local anesthesia, keeping a safety level of consciousness. It is an easy titratable drug, as controlled increasing amounts can be administered to the patient until the desired sedation level is reached. Once the administration of nitrous oxide is ceased, the patient returns to his/her previous state due to the quick reversibility of the gas effects (2-3).

At the same time, it is important to provide only the needed amount of drug to carry out the procedure, avoiding over sedation and thus favouring a positive dental experience (4).

The aim of this work was to study the physiological changes, the psychosedative and analgesic effects, as well as the non desired adverse effects of nitrous oxide-oxygen technique during a sedation procedure without dental treatment.

## Material and Methods

An observational investigation was performed in a convenient sample of dental students from the Valencia University Medical and Dental School. All subjects were healthy volunteers, with no contraindication for use of nitrous-oxide sedation. The study was approved by the Human Research Ethic Committee of the Valencia University.

Each subject took part in a single session, conducted by one experienced researcher with expertise in nitrous oxide administration technique and previously trained to perform the procedure in a standardized way. After signing the informed consent, a code number was assigned to each participant in order to guarantee data confidentiality. Volunteers were asked to fast an hour before the appointment.

-Procedure

Demographic data including age, sex, previous contact with nitrous oxide and hand dominance (left-handed, right-handed or ambidextrous) were recorded.

Pulse rate and hemoglobin oxygen saturation were registered using a portable finger pulse oximeter. Total length of procedure was calculated in minutes.

Throughout the sedation procedure and recovery period, the following parameters were registered.

-objective signs: trance-like expression, bright/fixed eyes, smile, voice changes, relaxed hands, limp legs, uncontrolled laughter, nistagmus, sweating, anxiety/nervousness, vomiting/nausea, incoherent/repeated words, consciousness/unconsciousness, hallucinations.

-symptoms referred by the subjects: dizziness, relax, sleepiness, heaviness/lightness, cold/warmth, tingling, feel good/bad, buzzing/vibration, different smell/taste, and hearing especially of distant sounds become more acute.

The recovery time was assessed, as well as other aspects such lethargy, slow/quick recovery or weak/firm legs.

Using the Johnson & Johnson One Touch Ultrasoft of LifeScan® for diabetics at level 5, a painful stimulus at constant intensity was generated to asses perception of pain during sedation. The pain stimuli were induced in the lateral part of the first phalanx in the dominant hand ring finger.

The pain experienced in each moment was scored following the Likert scale: 0 not annoying, 1 hardly annoying, 2 a bit annoying, 3 quite annoying, 4 very annoying.

Nitrous oxide/oxygen delivery equipment with appropriate scavenging system was used.

The administration of nitrous oxide was done in a standardized way with gradual increments of nitrous oxide. Signs and symptoms were recorded along the procedure and after oxygenation period. Pulse rate and hemoglobin oxygen saturation were monitored before nitrous oxide administration, after 30% N2O, after 50% N2O and after 100% oxygen. Perception of pain was assessed after 30% N2O, after 50% N2O and after 100% oxygen. The subject was never aware of type of gas he/she was inhaling, oxygen or oxygen with nitrous oxide, or its concentration.

Data were analyzed with SPSS 17.0 statistical software. ANOVA test was used to compare the pulse rate, hemoglobin oxygen saturation and the level of experienced pain in each study moment, as well as its comparison with the variables sex and previous contact with the gas. Pain was also compared with the variable dominance. Factorial correspondence analysis were carried out in order to determine any statistically significant (*p*< 0.05) relationships between signs and symptoms and the variables sex and previous contact with the gas.

## Results

101 subjects were enrolled in the study, 31 (30.7%) men and 70 (69.3 %) women. The mean age ± SD was 22.45 ± 3.53 (range 20-51 years). Only 7.9% of the sample (n=8) have had previous contact with nitrous oxide. Only 5.9% of subjects (n=6) were left-handed.

Total length of the procedure, was 23.4 minutes, with a standard deviation of ± 2.3 minutes.

The Physiologic parameters of hemoglobin oxygen saturation and pulse rate are illustrated in  Table 1.

**Table 1:**
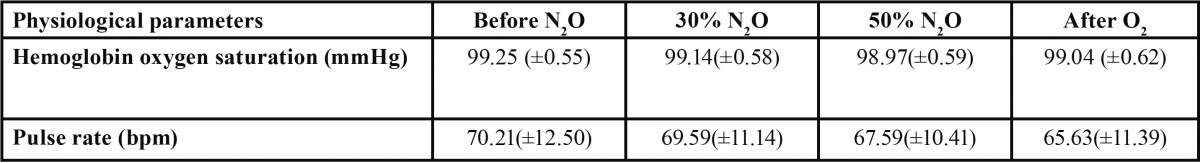
Mean value of physiologic parameters registered.

The frequencies of clinical signs in the total sample, from high to low frequency, were: bright or fixed eyes 99%, voice changes 98%, smile 91.1%, trance-like expression 70.3%, relaxed hands 59.4%, limp legs 55%, uncontrolled laughter 29.7%, anxiety/ nervousness 4%, nistagmus 1%, hallucinations 1% and nausea 1%.

The subjective effects more often reported were in descending order: tingling 98%, relax 91.1%, well-being 74.3%, heaviness 65.3%, warmth 46.5%, dizziness 41.6%, buzzing/vibration 37.6%, lightness 28.7%, smell/taste 17.8%, cold 13.9% and discomfort 8.9% .

The most significant data about the symptom frequency experienced by subjects in the recovery period were: relax 94.1%, quick recovery 75.2%, firm legs 67.3%, lethargy 51.5%, sleepiness 45.5%, tingling 37.6%, slow recovery 23.8%, dizziness 23.8%, weak legs 13.9% and headache 5.9%.

The percentage of subjects according to the level of pain experienced as not, hardly, bit, quite or very annoying in the three puncture moments is shown in  Table 2.

**Table 2:**
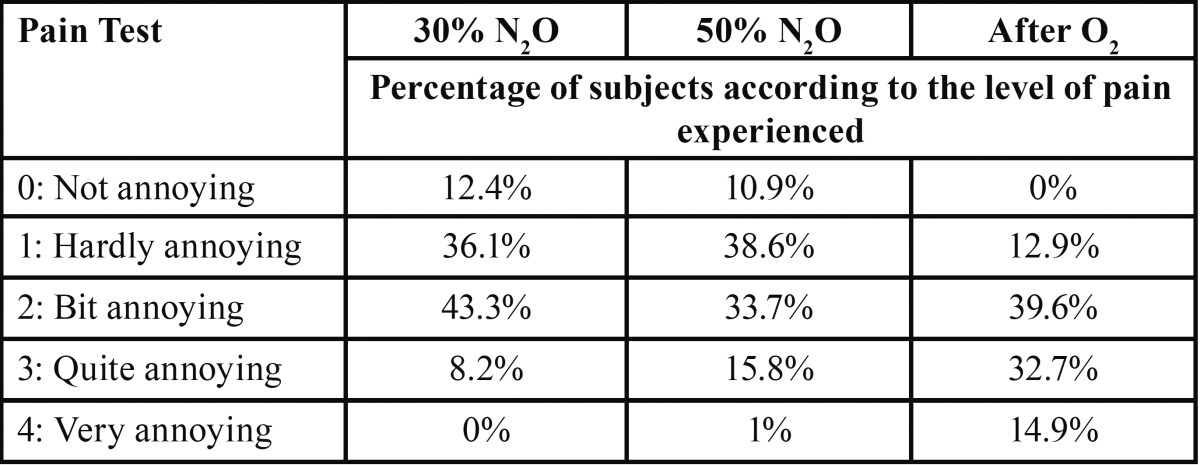
Percentage of subjects according to the level of pain experienced in the three puncture moments.

**Figure 1:**
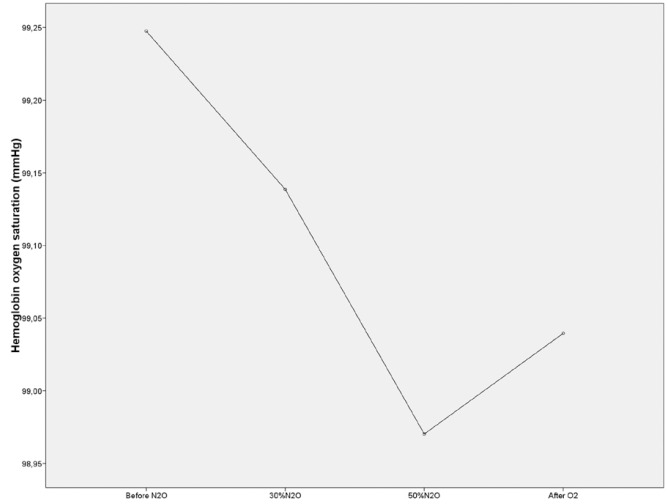
Hemoglobin oxygen saturation means in the four temporal moments.

**Figure 2:**
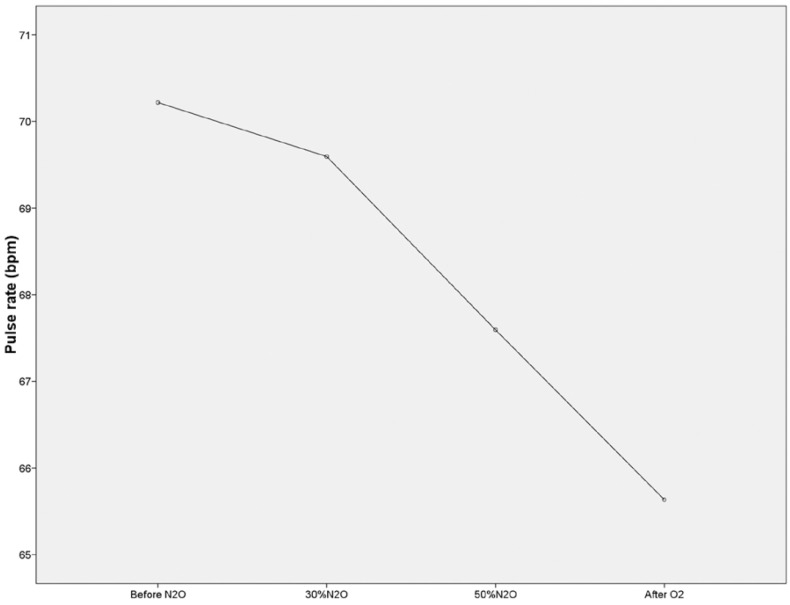
Pulse rate means in the four temporal moments.

An ANOVA was carried out in order to compare the hemoglobin oxygen saturation and heart rate values from the four temporal moments. The results from this analysis showed that there were statistically significant differences between hemoglobin oxygen saturation means in the four temporal moments (F3,300=7.804; *p* ≤ 0.05) figure 1 as well as heart rate (F3,300=12.922; *p* ≤0.05) (Fig. 2). A significant decrease in heart rate was observed throughout the procedure and even after the recovery period.

**Figure 3:**
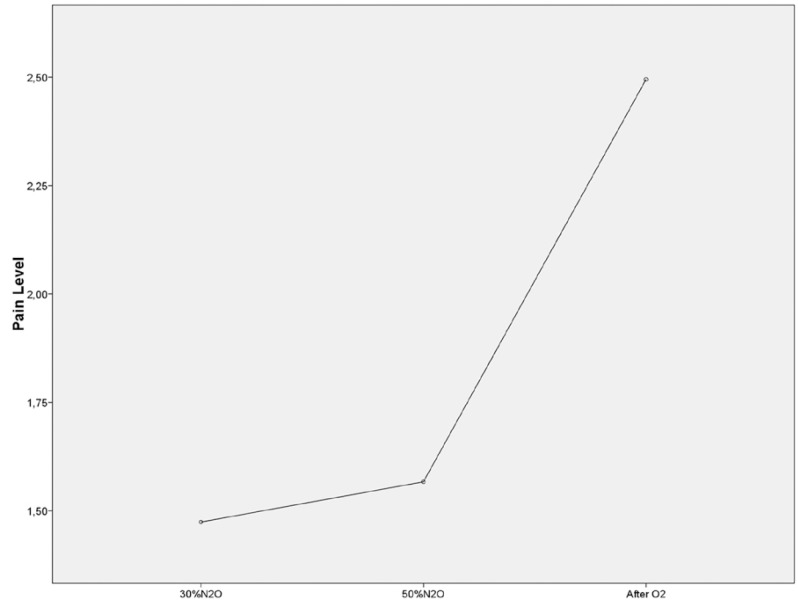
Pain level experienced at the three temporal moments.

We found statistically significant differences in the pain level experienced at the three temporal moments (F 2,192= 63.419; *p* ≤ 0.05) (Fig. 3).

No differences were detected between men and women referring to the effect of nitrous oxide in hemoglobin oxygen saturation (F3,297 = 0.828; *p* > 0.05), in pulse rate (F3,29 = 0.135; *p* > 0.05) or in pain level ( F2,190 = 0.236; *p* > 0.05), neither in the rest of parameters tested.

## Discussion

It is not the first time that university students (5-7) are enrolled to investigate the subjective effects of the gas, as they are considered to have the ability and experience to describe, communicate and fill questionnaires about it. In other works with the same objectives the subjects origin was different, due to a specific call (8-11) or collective (12-14), but they were always healthy volunteers, without any important medical or psychological problems who agreed to participate in studies of this kind. As in this research, most of the studies that aimed to identify the physiological changes and senses experienced with nitrous oxide sedation, were carried out in adults not receiving dental treatment. The effect of nitrous oxide on physiological parameters in patients under dental treatment could be masked by the reaction of the CNS to this treatment (15). Even in dental interventions without conscious sedation, stress or fear suffered in different moments could significantly alter these variables (16).

The final sample distribution with more women than men could have affected the results, but it is simply the expression of the current university context. Other previous studies with samples uniformly distributed between men and women did not analyze the possible relationship between sex and gas effects (7-12,14).

In this study two gas concentrations were chosen in order to standardize the procedure. The first one was of 30% as, according to the authors personal experience, it is a percentage where an appropriate sedation level has been generally achieved and where the subject experiences the pleasant senses associated with the gas, and thus the purpose was to corroborate it. The second was of 50%, as it is the maximum recommended concentration by the American Academy of Pediatric Dentistry (4) in order to avoid nitrous oxide adverse effects. No clear criteria have been found in the literature referring to the nitrous oxide concentration for the study of its effects in an isolated way. Some authors use a default nitrous oxide concentration (5,17), while others titrate the amount of administered gas until, based on their experience, the subject is considered to have reached an appropriate sedation level (7,18).

The variable hand dominance was included in order to perform always the pain test on the dominant motor hemisphere, and therefore avoid variables that have been related or not with the hemispheric cerebral dominance. Finally, only six subjects were found to be left-handed but the established protocol was applied to guarantee that this feature would not affect the results. In one study (6) the author applied electric stimulus on the first and third fingers of the participants dominant hand, although he does not clarify the rationale behind this choice.

In order to assess the psychosedative effects, analogical visual scales (8,9,19) have been used, as well as introspective reports (10,12,20) and questionnaires about psychodelic drug effects (8,11,13). However, in this work, we preferred to report information about a broad range of signs and symptoms mentioned before on the literature to determine which of them were identified more frequently with the senses experienced by students, with the aim of establishing a kind of list of terms usable in conscious sedation.

In order to analyze pain, a painful stimulus was generated with a puncture lancet for diabetics at the same point and with the same intensity in all the subjects. The lancet was calibrated at position 5, as the maximum position caused a very deep puncture which was too painful for participants. The puncture lancet for diabetics is a device easy to get, that generates a reproducible painful stimulus, as due to its calibration, the same intensity is always applied, helping in the comparative analysis of the results.

However, this way of generating painful stimulus has no precedents in the analyzed literature. Most studies are carried out in patients under dental treatment, therefore the studied painful stimulus is the application of local anesthesia or the pain inherent to the dental treatment, both conservative and surgical. In experimental situations, electric stimulus have been applied to patients in order to generate sensitivity or pain. These stimuli were generated with devices that produce electric shocks at different intensities, such as vitalometers (21) or electrostimulators (5,6). However, in the study carried out by Hogue, the use of the vitalometer was found not to be appropriate or conclusive.

There is neither a consensus on the election of the instruments for measuring the level of experienced pain. In this study, students were asked to score the pain according to the intensity in which they experienced it, in a Likert scale, being: 0 not annoying, 1 hardly annoying, 2 a bit annoying, 3 quite annoying and 4 very annoying. In spite of the difficulty of this aspect, as we are trying to objectify a subjective parameter, the followed procedure is broadly accepted (use of Likert scale) and allows the quantitative analysis of the results.

Hemoglobin oxygen saturation and heart rate recorded before the procedure were taken as basal values for each of the subjects, and allowed the detection of significant changes related with the gas effect. These changes reverted immediately in the case of oxygen saturation and persisted after recovery in the case of heart rate.

The significant decrease in hemoglobin oxygen saturation trough the procedure and the also significant recovery after the final oxygenation period, differ from the results of Primosh (18), Leelataweewud (22) and Wang (23), who did not observe statistically significant changes in these values, even though they used different methods and smaller samples.

However, the significant decrease in heart rate throughout the procedure and even after the recovery period was also detected by Hogue (21), Trieger (5), Primosh (24), Allison (14), Nathan (25), Mueller (26) and Major (27). In contrast, Roberts (15) found a light pulse increase ten minutes after starting the procedure, coinciding with the administration of local anesthesia and after the dental treatment performed in children. The significant pulse decrease could be considered as a sign related with sedation, but not characteristic of the desired sedation level, as it decreases progressively even through oxygenation.

The most common presented signs were: fixed and/or bright eyes, voice changes, consciousness, smile, trance-like expression, relaxed hands and legs. Trance-like expression, smile or relaxed hands and legs were also the signs found with a higher frequency by Houpt (20), although his work was carried out on a child population and under dental treatment conditions. Nevertheless, it is the first time that the sign of fixed bright eyes is mentioned. It can be considered a characteristic aspect of the trance facial expression, which in our opinion shows that the subject is sedated.

As in this work, various authors corroborate the maintenance of the consciousness level of the subjects receiving the gas mix (15,28), which confirms the safety of its use.

In accordance with other authors, the symptoms most frequently experienced by the subjects in this study were: tingling (12,20,28), relax (6,23), well-being (8,20), heaviness (20,23) and heat (20,28). It is also remarkable, that when we ask the subjects about specific senses, we can sometimes be influencing them. However, the obtained results show that students could clearly identify experienced and non experienced senses.

Nausea and vomiting are the adverse effects more often associated with nitrous oxide conscious sedation. A higher incidence is noted with longer administration of nitrous oxide/oxygen, fluctuations and increased concentrations of nitrous oxide. Fasting is not required for patients undergoing nitrous oxide analgesia/anxiolysis. The practitioner, however, may recommend that only a light meal be consumed in the two hours prior to the administration of nitrous oxide (4). Therefore, in this study subjects were asked to fast an hour before the appointment. Only 8.9% presented discomfort and 1% had nausea, but without vomiting, thus the frequency of nausea associated with sedation was found to be very low.

Most participants in the study described the recovery as quick and immediate, accordingly with previous reports in the literature (8,29). Only 5.9% of subjects experienced headache that can be avoided by administering 100 percent oxygen after nitrous oxide has been discontinued, as reported in other studies (21,29).

In previous investigations analyzing the analgesic effect of the gas without performing any dental treatment, electric stimulus were used, observing a higher tolerance to voltage increase after nitrous oxide inhalation (5,6,23). Nevertheless, the situation is more complex when there is dental pain. It has been said that the “relative” analgesia experienced by patients undergoing dental treatment with nitrous oxide sedation is more directly related to a relief of anxiety, secondary to the subjective changes induced by the gas as well as the placebo effects developed between doctor and patient (5). The results of our work show that the pain sense experienced by subjects was higher when they were no longer under nitrous oxide effects. However, it is important to point out that with 50% nitrous oxide, a higher percentage of patients reported the experienced sense as quite or very annoying, presenting a progressive sensitization, regardless of the nitrous oxide concentration, which should be considered in clinical procedures. It has to be noted that repeating the procedure three times in the same subject could have produced a sensitization to pain showed in the highest nitrous oxide concentrations.

Our results confirm that nitrous oxide, as unique agent, possesses an analgesic effect, and support the hypothesis that its effect does not increase with the dose, rather the analgesia is produced at a concentration of 30% nitrous oxide. This finding has a direct applicability in the clinical nitrous oxide administration procedure.

This results differ from those presented by Trieger (5), who concluded that the analgesic effect is related with the dose of nitrous oxide. Nonetheless, further research on this aspect would be required, as although in both cases the studies were performed with dentistry students, in his work the sample size was smaller and the nitrous oxide concentrations used were very high, thus increasing the possibility of reaching an anesthetic stage where subjects would not respond to the painful stimulus.

As a conclusion, it was observed in this study that nitrous oxide causes a significant decrease in oxygen saturation and heart rate in the tested group, which does not endanger the safety of the procedure. The hemoglobin oxygen saturation was restored during the recovery period while heart rate decreased trough and after the procedure. 

Symptoms such as “warmth”, “tingling” and “weakness”, and signs such as “bright eyes”, “voice changes”, “smile” and “trance-like expression” can help the practitioner to determine if the subject is reaching an appropriate sedation level, thus avoiding over sedation.

The analgesic effect of nitrous oxide is confirmed, supporting the hypothesis that this effect does not increase with a higher gas dose.

In all sessions, all subjects kept an appropriate level of consciousness, confirming the safety of this technique at the used concentrations.
